# Hot-Air Treatment

**Published:** 1900-08-25

**Authors:** 


### HOT-AIR TREATMENT.

Dr. David Walsh[1] discusses the utility of hot air treatment in various gouty and other affections. The point of greatest interest which he makes is that the application of hot, dry air, even to a comparatively small portion of the body, has a very widespread influence. This has already been shown by other observers, but it is worthy of emphasis because it is by no means improbable that much of the benefit derived by many patients from this line of treatment has more to do with the efforts made by the whole organism to protect the part exposed from injury by the excessive heat than from any direct influence of the heat itself on the part in question. Dr. Walsh says that after the part has been exposed to hot air for a varying period there is free acid sweating, together with increased frequency of pulse, while the body temperature is raised 2 deg. or 3 deg. Fahr. In addition to this there is often an immediate and striking relief of pain, and the range of movement in stiffened joints is increased. As showing how widespread the influence of the local application of heat may be, he notes that pain in a foot may be relieved by placing an arm in the apparatus, and a stiff elbow may move more freely after local treatment of a leg. Notwithstanding the free action of the skin during the application, an increased elimination by the kidneys seems also to take place, and Dr. Chrétien, of Paris, has shown that while the hot-air baths are being taken the daily excretion of uric acid is distinctly increased. Dr. Walsh says that generally speaking the hot-air treatment is likely to do good in (1) painful nervous affections; (2) many painful and stiffened joints; (3) other diseases, such as anæmia and Bright's disease; (4) eczematous skin troubles; and (5) arthritic diseases. Clearly there are many openings for the use of hot air, and it ought not to be difficult of application.

[1] Lancet, Aug. 18.

